# Acute Upper Airway Disease in Children With the Omicron (B.1.1.529) Variant of SARS-CoV-2—A Report From the US National COVID Cohort Collaborative

**DOI:** 10.1001/jamapediatrics.2022.1110

**Published:** 2022-04-15

**Authors:** Blake Martin, Peter E. DeWitt, Seth Russell, L. Nelson Sanchez-Pinto, Melissa A. Haendel, Richard Moffitt, Tellen D. Bennett

**Affiliations:** 1 Section of Critical Care Medicine, Department of Pediatrics, University of Colorado School of Medicine, Aurora; 2 Section of Informatics and Data Science, Department of Pediatrics, University of Colorado School of Medicine, Aurora; 3 Division of Critical Care, Department of Pediatrics, Northwestern University Feinberg School of Medicine, Chicago, Illinois; 4 Department of Biochemistry and Molecular Genetics and the Center for Health Artificial Intelligence, University of Colorado School of Medicine, Aurora; 5 Department of Biomedical Informatics, Stony Brook University, Stony Brook, New York

## Abstract

This cohort study uses data from the US National COVID Cohort Collaborative to evaluate upper airway infections in children during the surge of the Omicron (B.1.1.529) variant of SARS-CoV-2 in the US.

SARS-CoV-2 can cause severe pediatric disease, including acute COVID-19 and multisystem inflammatory syndrome.^1^ Published reports associating SARS-CoV-2 with upper airway infection (UAI), such as laryngotracheobronchitis (croup), have been limited to small case series.^2^ Although noncoronaviruses, including parainfluenza and respiratory syncytial virus, most frequently cause UAI, coronaviruses (eg, type NL63) are also commonly implicated. Young children are especially vulnerable to UAI given their small and relatively collapsible airways.

The Omicron (B.1.1.529) strain of SARS-CoV-2 became dominant in the US the week ending December 25, 2021.^3^ Omicron is known to cause lower severity disease than the Delta (B.1.617.2) variant. This may be because Omicron replicates less efficiently in lung parenchyma and more efficiently in the conducting airways.^4^ We conducted this retrospective cohort study to determine if cases of UAI among children increased when Omicron became the dominant SARS-CoV-2 variant in the US.

## Methods

We leveraged the US National COVID Cohort Collaborative (N3C)^5^ and a pipeline we built for a National Institutes of Health–funded pediatric COVID-19 dashboard^6^ to conduct this study. Among children in N3C younger than 19 years with a positive SARS-CoV-2 test result (polymerase chain reaction, antigen, or antibody), we identified those with a UAI diagnosis (eTable in the Supplement). We included bacterial tracheitis because it can be difficult to distinguish from and can be a complication of viral croup. We compared demographic, comorbidity, and clinical outcome variables between patients from the pre-Omicron (March 1, 2020, to December 25, 2021) and Omicron (December 26, 2021, to February 17, 2022) periods. We used χ^2^ and Fisher exact tests for categorical variable comparisons and the Mood median and *t* tests for continuous variable comparisons. Race and ethnicity were identified from N3C site electronic health record data and included to aid with identification of variables associated with increased risk of UAI among children with SARS-CoV-2. Each N3C site determines race and ethnicity at its discretion. We used linear regression to determine the change over time in percentage of children with UAI among children hospitalized with SARS-CoV-2. The N3C Data Enclave, data transfer from sites to N3C, and this analysis were approved under separate institutional review board protocols as documented elsewhere.^1^ The N3C Data Enclave is approved under the authority of the National Institutes of Health Institutional Review Board. Each participating N3C site maintains an institutional review board–approved data transfer agreement. The analyses in this article were approved by the institutional review boards of the study investigators with data access, which includes a waiver of informed consent.

## Results

The February 17, 2022, N3C data release contains 18 849 children hospitalized with SARS-CoV-2, 384 of whom (2.0%) had UAIs (Table). Severe disease (defined as requiring invasive ventilation, vasopressors, or extracorporeal membrane oxygenation or death) occurred in 81 children (21%).

**Table.** Characteristics and Outcomes of Hospitalized Children With Upper Airway Infection (UAI) and SARS-CoV-2 During the Pre-Omicron and Omicron Periods

Variable	Hospitalized UAI cases, No./Total No. (%); SE			*P* value
	All (N = 384)	Pre-Omicron (n = 206)	Omicron (n = 178)	
% Children hospitalized with SARS-CoV-2 found to have UAI	384/18 849 (2.0); 0.2	206/14 473 (1.4); 0.2	178/4376 (4.1); 0.6	<.001
Sex				.72
Female	132/384 (34.4); 4.9	79/206 (38.3); 6.8	53/178 (29.8); 6.9	
Male	252/384 (65.6); 4.9	127/206 (61.7); 6.8	125/178 (70.2); 6.9	
Age, mean (SD), y	3.3 (3.8)	4.4 (4.5)	2.1 (2.1)	<.001
Ethnicity^a^				<.001
Hispanic or Latino	102/384 (26.6); 4.5	39/206 (18.9); 5.6	63/178 (35.4); 7.2	
Not Hispanic or Latino	255/384 (66.4); 4.8	152/206 (73.8); 6.2	103/1785 (7.9); 7.5	
Missing/unknown	27/384 (7.0); 2.7	<20 (<9.7)	<20 (<11)	
Race^a^				<.001
Asian	<20 (<5.2)	<20 (<9.7)	<20 (<11)	
Black or African American	50/384 (13.0); 3.5	40/206 (19.4); 5.4^b^	<20 (<11)	
Native Hawaiian or other Pacific Islander	<20 (<5.2)	<20 (<9.7)	<20 (<11)	
White	222/384 (57.8); 5.0	114/206 (55.3); 7.0	108/178 (60.7); 7.4	
Other^c^	89/384 (23.2); 4.3	46/206 (22.3); 5.9	43/178 (24.2); 6.5	
Missing/unknown	<20 (<5.2)	<20 (<9.7)	<20 (<11)	
Comorbidities				
Known BMI	91/384 (23.7); 4.4	71/206 (34.5); 6.7	201/178 (11.2); 4.9	<.001
Obese (BMI ≥95th percentile)^d^	<20 (<5.2)	<20 (<9.7)	<20 (<11)	NA
Diabetes (type 1 or 2)	<20 (<5.2)	<20 (<9.7)	<20 (<11)	NA
Asthma	42/384 (10.9); 3.3	40/206 (19.4); 5.4^b^	<20 (<11)	.41
Medications received				
Dexamethasone	114/384 (29.7); 4.7	75/206 (36.4); 6.8	39/178 (21.9); 6.3	<.001
Systemic antibiotic	100/384 (26.0); 4.5	80/206 (38.8); 6.9^b^	<20 (<11)	<.001
SARS-CoV-2 severity^e^				<.001
Moderate	303/384 (78.9); 4.2	131/206 (63.6); 6.8	172/178 (96.6); 3.1	
Severe	81/384 (21.1); 4.2	80/206 (38.8); 6.8^b^	<20 (<11)	
Mechanical ventilation	76/384 (19.8); 4.1	70/206 (34.0); 6.7^b^	<20 (<11)	
Vasoactive inotropes	34/384 (8.9); 3.0	30/206 (14.6); 5.2^b^	<20 (<11)	

Abbreviations: BMI, body mass index; NA, not applicable; SE, standard error.

^a^ The method by which each N3C site determines and stores race and ethnicity information is at the discretion of each participating health care site. Race and ethnicity variables were included in this analysis to help identify factors associated with development of UAI among children hospitalized with SARS-CoV-2.

^b^ Result rounded to the nearest 10 to avoid exposure of cell values under 20 (as per N3C policy). Percentages are represented as if n = 20.

^c^ Includes patients with a race value reported to the N3C by the health care site of other, other race, more than 1 race, or multiple race.

^d^ BMI calculated as per the US Centers for Disease Control and Prevention guidelines with obesity defined as any child 2 years and older with a BMI ≥95th percentile for age and sex. Percentages reported in the obese row represent the percentage of patients with a known BMI who had a BMI greater than 95th percentile for age and sex.

^e^ Severe disease includes children requiring invasive ventilation, vasoactive inotropes, or extracorporeal membrane oxygenation support or who died, whereas moderate disease includes hospitalized children without any of these. The number of patients who required extracorporeal membrane oxygenation support and the number of patients who died were both <20 and are not shown.

SARS-CoV-2–positive UAI rates have increased with progression from the pre-Omicron to Omicron periods (206 of 14 473 [1.5%] vs 178 of 4376 [4.1%], respectively; *P* < .001) (Figure), with 178 of 384 cases (46%) occurring during the Omicron period. Children with UAIs during the Omicron period were more likely to be younger and Hispanic or Latino and less likely to receive dexamethasone or develop severe disease compared with those in the pre-Omicron period. Lastly, the proportion of children with a pediatric complex chronic condition was not significantly different in the pre-Omicron period compared with the Omicron period (74 of 206 [36%] vs 39 of 178 [22%], respectively; P = 0.54).

**Figure.**
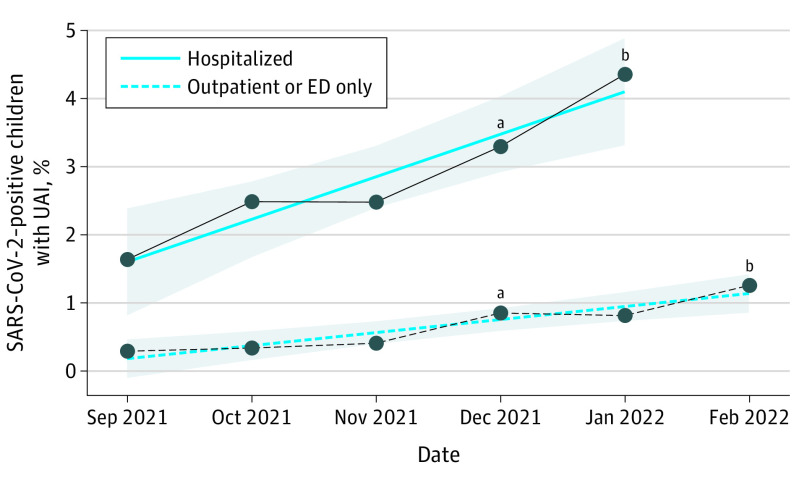
SARS-CoV-2–Positive Children With Upper Airway Infection (UAI) The figure shows the percentage of pediatric SARS-CoV-2 cases per month among inpatient (solid line) and outpatient/emergency department (dotted line) encounters with a diagnosis of UAI within the National COVID Cohort Collaborative (N3C) February 17, 2022, data release. Per N3C policy, only data points in which the group (inpatient or outpatient and emergency department [ED]) had at least 20 patients are shown to prevent exposure of patient counts fewer than 20. Prior months are not shown given patient counts of fewer than 20 per month within the inpatient group before September 2021. Hospitalizations in February 2022 were fewer than 20 and are not shown. ^a^The percentage of sequenced SARS-CoV-2 samples found to be the Omicron strain among samples from weekly variant testing by the US Centers for Disease Control and Prevention COVID-19 Data Tracker^3^ increased from 0.6% during the week ending December 4, 2021, to 89.2% during the week ending January 1, 2022. ^b^Linear regression identified the rate of change per month in SARS-CoV-2–positive children with a UAI diagnosis as 0.6% (standard error, 0.1%; *P* = .008) among hospitalized cases (solid line) and 0.2% (standard error, 0.03%; *P* = .005) among outpatient and emergency department cases (dotted line). Shaded regions indicate 95% CIs.

## Discussion

SARS-CoV-2–positive pediatric UAI rates increased during the Omicron surge. More than one-fifth of children hospitalized with SARS-CoV-2 and UAI developed severe disease. Given the high proportion of UAI cases during the Omicron period, these results appear to support recent mechanistic reports. A limitation of this analysis is that diagnosis codes will only be present for completed encounters; as such, children who are still hospitalized are not represented, and the frequency of severe disease observed in the Omicron period may be an underestimate.

Children with severe UAI are at risk of cardiac arrest from rapid-onset upper airway obstruction. They may require therapies typically provided in intensive care units, including frequent administration of nebulized racemic epinephrine, helium-oxygen mixtures, and intubation. While the rate of SARS-CoV-2 pediatric UAI is not overwhelmingly high, understanding this new clinical phenotype and the potential for acute upper airway obstruction may help guide therapeutic decision-making.

## Notes

Supplement. eTable. Included diagnoses
