# Supplementary material for: Acute Upper Airway Disease in Children With the Omicron (B.1.1.529) Variant of SARS-CoV-2—A Report From the US National COVID Cohort Collaborative
Source: JAMA Pediatr. 2022 Apr 15;176(8):819–21. doi: 10.1001/jamapediatrics.2022.1110 (PMC9012983; doi:10.1001/jamapediatrics.2022.1110)
Supplement: Supplement. — eTable. Included diagnoses [file jamapediatr-e221110-s001.pdf]

Supplemental Online Content

Martin B, DeWitt PE, Russell S, et al. Acute upper airway disease in children with the Omicron (B.1.1.529) variant of SARS-CoV-2—a report from the US National COVID Cohort Collaborative. *JAMA Pediatr*. Published online April 15, 2022. doi:10.1001/jamapediatrics.2022.1110

eTable. Included diagnoses

This supplemental material has been provided by the authors to give readers additional information about their work.

© 2022 Martin B et al. *JAMA Pediatrics.*

eTable. Included diagnoses

Concept Name	Concept Code	Concept Id	Domain Id	Vocabulary Id	Concept Class Id	Standard Concept
Acute bacterial epiglottitis	21060003	4050381	Condition	SNOMED	Clinical Finding	S
Acute epiglottitis	29608009	4105773	Condition	SNOMED	Clinical Finding	S
Acute epiglottitis (non-streptococcal)	266337001	4141503	Condition	SNOMED	Clinical Finding	S
Acute epiglottitis with obstruction	222008	433513	Condition	SNOMED	Clinical Finding	S
Acute epiglottitis without obstruction	49908003	435840	Condition	SNOMED	Clinical Finding	S
Acute fibrinous laryngotracheobronchitis	275495004	4170141	Condition	SNOMED	Clinical Finding	S
Acute infective tracheobronchitis	312400008	4208810	Condition	SNOMED	Clinical Finding	S
Acute laryngitis and/or tracheitis	276443001	4173027	Condition	SNOMED	Clinical Finding	S
Acute toxic tracheobronchitis	233799004	4121456	Condition	SNOMED	Clinical Finding	S
Acute tracheitis	26650005	437903	Condition	SNOMED	Clinical Finding	S
Acute tracheitis with obstruction	8519009	255566	Condition	SNOMED	Clinical Finding	S
Acute tracheitis without obstruction	64369009	4273095	Condition	SNOMED	Clinical Finding	S
Acute tracheobronchitis	35301006	4148204	Condition	SNOMED	Clinical Finding	S
Adenoviral laryngotracheobronchitis	71255007	4322815	Condition	SNOMED	Clinical Finding	S
Adult acute epiglottitis and supraglottitis	232433008	4051610	Condition	SNOMED	Clinical Finding	S
Catarrhal tracheitis	50211006	4173594	Condition	SNOMED	Clinical Finding	S
Croup	71186008	260134	Condition	SNOMED	Clinical Finding	S
Epiglottitis	80384002	4214374	Condition	SNOMED	Clinical Finding	S
Haemophilus influenzae epiglottitis	58576005	4242412	Condition	SNOMED	Clinical Finding	S
Haemophilus influenzae laryngotracheobronchitis	73414003	4249017	Condition	SNOMED	Clinical Finding	S
Infection causing tracheitis in neonate	735740009	42536748	Condition	SNOMED	Clinical Finding	S
Infectious disorder of trachea	233785003	4121450	Condition	SNOMED	Clinical Finding	S

© 2022 Martin B et al. *JAMA Pediatrics.*

Laryngeal obstruction due to supraglottitis	3.6491E+13	46270516	Condition	SNOMED	Clinical Finding	S
Laryngotracheobronchitis	85915003	4312602	Condition	SNOMED	Clinical Finding	S
Parainfluenza virus laryngotracheobronchitis	35377009	4146838	Condition	SNOMED	Clinical Finding	S
Pediatric acute epiglottitis and supraglottitis	232432003	4048195	Condition	SNOMED	Clinical Finding	S
Recurrent croup	2.2951E+13	46270340	Condition	SNOMED	Clinical Finding	S
Respiratory syncytial virus laryngotracheobronchitis	72204002	4218289	Condition	SNOMED	Clinical Finding	S
Supraglottitis	709663002	46271527	Condition	SNOMED	Clinical Finding	S
Tracheitis	62994001	4270490	Condition	SNOMED	Clinical Finding	S
Tracheobronchitis	13617004	4028970	Condition	SNOMED	Clinical Finding	S
Viral epiglottitis	70976000	4321233	Condition	SNOMED	Clinical Finding	S
Viral tracheitis	66011008	4278083	Condition	SNOMED	Clinical Finding	S

© 2022 Martin B et al. *JAMA Pediatrics.*
